# Does the Combined Use of Aspirin and Immunotherapy Result in Better Outcomes in Non-Small Cell Lung Cancer Than Immunotherapy Alone?

**DOI:** 10.7759/cureus.25891

**Published:** 2022-06-13

**Authors:** Mina Aiad, Ali Tahir, Kayla Fresco, Zarian Prenatt, Karla Ramos-Feliciano, Jasmit Walia, Jill Stoltzfus, Heidar J Albandar

**Affiliations:** 1 Internal Medicine, St. Luke's University Health Network, Bethlehem, USA; 2 Internal Medicine, Lewis Katz School of Medicine at Temple University, Philadelphia, USA; 3 Hematology and Medical Oncology, St. Luke's University Health Network, Bethlehem, USA

**Keywords:** recist criteria, cancer survival, ecog (eastern cooperative oncology group), non-small cell lung cancer (nsclc), cyclooxygenase inhibitors, prostaglandin e2, aspirin therapy, programmed death-ligand 1, pd-1 inhibitors, cancer immunotherapy

## Abstract

Introduction: Immunotherapy works by stimulating the immune system against cancer cells. Resistance to immunotherapy represents a significant challenge in the field of medical oncology. The mechanisms by which cancer cells evade immunotherapy are not well understood. Prior research suggested overexpression of prostaglandin E-2 (PGE-2) by cancer cells, which bind to EP-2 and EP-4 receptors on the tumor-specific cytotoxic T-lymphocytes (CTLs) and suppress their anticancer role. This immunosuppressive effect is involved in evading the programmed cell death-1 (PD-1)/programmed death-ligand 1 (PD-L1) blockade of immunotherapy, which fuels cancer cell growth and recurrence. Studies found that combining PGE-2 blockade and a PD-1 signaling inhibitor helped promote the anticancer immunity cells. If confirmed in a clinical setting, the above in vitro findings could be of great clinical significance.

Methods: Given that aspirin (ASA) blocks PGE-2 production, this work aimed to evaluate whether ASA use with immunotherapy results in better outcomes than immunotherapy alone. We performed a retrospective chart review of 500 non-small cell lung cancer (NSCLC) patients aged 21 years or older treated with PD-1 and/or PD-L1 directed immunotherapy at St. Luke’s University Health Network between July 2015 and July 2021. Relevant patient, disease, and treatment-related variables were collected, including ASA use (≥ 81 mg daily) and the type of immunotherapy. Bivariate analyses were conducted to determine which variables to include in a multivariable model.

The four primary outcomes included survival at 18-months, both after diagnosis and starting immunotherapy, achieving complete remission (CR), and having a progressive disease (PD), as defined by RECIST (Response Evaluation Criteria in Solid Tumors) criteria. Secondary outcomes included therapy-related toxicities and complications in the different treatment groups.

Results: After bivariate analysis, no statistical significance was found for a difference in 18-month survival between ASA and non-ASA groups (50.3% vs 49.7%, p-value = 0.79). ASA with PD-L1 inhibitor showed a trend towards a higher likelihood of achieving CR [adjusted odds ratio (AOR) 1.85] with a p-value close to statistical significance (0.06). However, ASA with PD-L1 showed high statistical significance as an independent variable associated with a decreased likelihood of having PD (AOR 0.44, p < 0.001). These findings suggest that NSCLC patients receiving PD-L1 inhibitors could benefit more from daily ASA than patients treated with PD-1 inhibitors. Our study emphasizes using the Eastern Cooperative Oncology Group (ECOG) scoring of the performance status (PS) in NSCLC patients. Poorer PS was associated with lower survival, decreased likelihood of CR, and more PD. Other variables associated with worse outcomes were advanced cancer stage at diagnosis and male gender. Low-PD-L1 expression in NSCLC was associated with an increased likelihood of survival; this could be of clinical significance, especially with previous studies suggesting better outcomes of using ASA in PD-L1 low tumors.

Conclusion: These findings suggest that daily ASA use with PD-L1 inhibitors is associated with more favorable outcomes in NSCLC. More studies are needed to investigate further the potential benefits vs. risks of using ASA with different immunotherapies and the other possible variables affecting treatment outcomes.

## Introduction

Immunotherapy is considered a revolutionary cancer treatment. As more studies suggest its favorable outcomes and synergistic effects to other cancer therapies, it is being used more commonly and is becoming one of many cancer mainstream treatments [1].

The programmed cell death-1 (PD-1) and programmed death ligand-1 (PD-L1) inhibitors are immune checkpoint inhibitors (ICPs) that block the cancer cell's ability to evade the tumor-specific cytotoxic T lymphocytes (CTLs). The body's "normal" cells express surface PD-L1 proteins, which bind to the PD-1 receptors expressed on the CTLs [2]. Such binding keeps CTLs from attacking the PD-L1-containing cells. Some cancer cells overexpress PD-L1 proteins to "inactivate" the anti-cancer T-cells and evade the natural immune response. PD-1/ PD-L1 inhibitors block this negative CTLs regulation and enhance the number and effectiveness of CTLs against cancer cells [3].

Prior in vitro studies suggest that some cancer cells produce prostaglandin E-2 (PGE-2), which induces an inflammatory process promoting their proliferation and metastasis [3]. The binding of cancer-induced PGE-2 to its receptors EP2 and EP4 on CTLs results in their "inactivation" and immune evasion [4]. Therefore, blocking the PGE-2 production or its negative CTLs regulatory pathway was thought to enhance the immune cells' role against cancer cells [5]. Cyclooxygenase-2 (COX-2) is a rate-limiting enzyme of PGE2 production from arachidonic acid. Prior research found that the simultaneous delivery of celecoxib (COX-2 inhibitor) and PD-1 inhibitor to tumor-bearing mice enhanced T-cell immunity against cancer cells and decreased tumor-induced inflammation and angiogenesis [6]. Similarly, another study found that inhibition of microsomal prostaglandin E synthase-1 (mPGES-1), an enzyme involved in PGE-2 production, inhibited prostate cancer cells' spheroid growth [7].

Besides in vitro research, many human studies suggest the potential benefits of using aspirin (acetylsalicylic acid or ASA), a nonselective COX-inhibitor non-steroidal anti-inflammatory drug (NSAID), in lowering risk as well as improving outcomes of some cancers [8]. A large meta-analysis of controlled and observational studies concluded that ASA use, especially in high doses for more than 10 years, is associated with a lower incidence of colonic adenoma and colorectal cancer (CRC) [9]. Another meta-analysis of 51 randomized controlled trials suggests that ASA use is associated with decreased long-term death from all cancers, including CRC [10]. Furthermore, a meta-analysis of observational studies suggested a significant decrease in breast cancer risk with ASA use [11].

Given the above findings of the potential role of inhibiting PGE-2 production along with PD-1/PD-L1 blockade in enhancing immunity against cancer cells, our study aimed to determine whether there is a synergetic effect when combining ASA with immunotherapy in the treatment of non-small cell lung cancer (NSCLC) patients.

## Materials and methods

Patients aged 18 years or older with NSCLC diagnosed between July 2015 and July 2021 who received at least one dose of immunotherapy at St. Luke’s University Health Network were identified on an IRB-approved protocol (IRB 00002757 issued approval SLIR 2021-121). We performed a retrospective chart review to collect relevant data, classified as patient-, disease- and treatment-related variables, and the outcomes. Patient-related variables included gender, race, age, pre-existing chronic obstructive pulmonary disease (COPD), and performance status (PS) at NSCLC diagnosis. COPD severity was determined according to the spirometry guidelines of the Global Initiative for Chronic Obstructive Lung Disease (GOLD), and the PS was determined based on ECOG criteria.

Disease-related variables included the subtype of NSCLC, PD-L1 expression, and cancer stage at diagnosis. Treatment-related variables included the type of immunotherapy used [PD-1, PD-L1, or CTLA-4 (Cytotoxic T Lymphocyte Associated Protein 4) inhibitor], whether the patient received concurrent chemotherapy, the use of ASA, and its indication. The ASA use was identified as a minimum of 81 mg daily and it was classified into four categories: non-user (identified as having never been on daily ASA), prior-user (history of daily ASA before but not while receiving immunotherapy), concurrent-user (started after cancer diagnosis and while receiving immunotherapy), or ASA-user (defined as long-term use of ASA, both prior to and along with immunotherapy).

Given that some patients used immunotherapy as a second-line treatment and had a relatively long duration (few months) between diagnosis and starting immunotherapy, we decided to evaluate survival duration after both diagnosis and starting immunotherapy. As an indicator of the response to treatment(s), the most recent Response Evaluation Criteria in Solid Tumors (RECIST) v1.1 is determined based on available imaging (computerized and positron emission tomography scans) and health providers’ documentation. We chose to use RECIST 1.1 over its modified immune-based version (iRECIST) mainly because we reviewed charts that predated the 2017 adoption of the iRECIST, and thus we avoid reclassifying some of the already evaluated responses based on RECIST 1.1. As applicable, the duration of complete remission (CR) was determined and classified into intervals (<6 months, 6-12 months, 13-24 months, 25-36 months, 37-60 months, or > 5 years). As secondary outcomes, information about whether the patients had therapy-induced toxicities or complications were classified into systematic groups: pneumonitis, hematologic, gastroenteric, thyroid disease, or others (including integumentary, hypertension, hepatic, neuropathy, not listed, or multiple toxicities).

After adjusting for relevant patients' demographic and clinical variables, we constructed separate multivariable direct logistic regression models to determine the independent contribution of ASA combined with immunotherapy to the primary outcomes. We aimed to evaluate the effect of combined ASA/aggregated immunotherapy (defined as ASA use with any type of immunotherapy) and combined ASA/specific type of immunotherapy (PD-1 vs. PD-L1 inhibitors). Given the limited subgroup sample for patients treated with both PD-1 and PD-L1 immunotherapies (n = 15; two of them received PD-L1, PD-1, and CTLA-4 inhibitors), this category was excluded from our analysis. Similarly, only one patient was treated with only CTLA-4 inhibitor immunotherapy and was excluded as well.

Before regression modeling, separate bivariate analyses were done comparing each potential covariate and our outcomes to determine which variables were best suited to multivariate modeling at p < 0.20 for three of the four outcomes. For CR, we chose a more conservative p-value of < 0.10 to help ensure adequate subgroup sample sizes for modeling, given our limited number of events (n = 83). In addition to treatment with ASA and immunotherapy (both aggregated and sorted by specific type), our potential covariates, selected based on previous research and clinical considerations, included the following: age at the time of lung cancer diagnosis, gender, race, pre-existing COPD, ECOG score, type of lung cancer, stage of lung cancer at diagnosis, PDL-1 expression, and simultaneous chemotherapy with immunotherapy.

We also assessed the presence of outliers and influential data points before regression modeling. Across the four primary outcomes, outlier values ranged from 0.8% to 6.2%, with no influential data points based on examination of the normalized residuals, Cook’s D, and leverage statistics. Therefore, we retained all patients in our different models to ensure the broadest possible external generalizability. We reported the omnibus chi-square statistic and the Hosmer-Lemeshow goodness-of-fit statistic to ascertain model fit. We present adjusted odds ratio (AOR) and 95% confidence interval (CIs) for each covariate.

Our secondary outcomes were the presence of pneumonitis, hematological complications, gastroenteric complications, and other complications. We analyzed these outcomes using separate chi-square tests, first for combined ASA/aggregated immunotherapy, and then for combined ASA/specific type of immunotherapy (PD-1 vs. PD-L1).

We used SPSS version 28 (Armonk, NY: IBM Corp.) to analyze our data, with p < 0.05 denoting statistical significance for all outcomes and no adjustment for the multiple comparisons. In addition, study data were collected and managed using REDCap electronic data capture tools hosted at St. Luke’s University Health Network.

## Results

**Figure 1:**
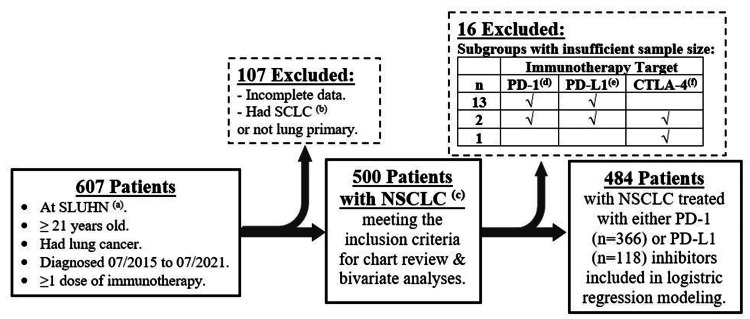
A flowchart of our retrospective study design. (a) SLUHN, St. Luke's University Health Network; (b) SCLC, small cell lung cancer; (c) NSCLC, non-small cell lung cancer; (d) PD-1, programmed cell death-1; (e) PD-L1, programmed death-ligand 1; (f) CTLA-4, cytotoxic T-lymphocyte-associated antigen 4.

There were 500 patients in our sample: 112 patients aged 21-60 years (22.4%); 274 patients aged 61-75 years (54.8%); 114 patients aged > 75 years (22.8%); 220 females (44.0%), 280 males (56.0%); 461 Caucasian (92.2%), and 39 non-Caucasian (7.8%). A total of 484 patients had complete data for analysis and were, therefore, included in our logistic regression modeling (Figure 1).

Table 1 displays the survival at 18-months after NSCLC diagnosis. The following covariates were included in the multivariable regression model (p < 0.20): pre-existing COPD, ECOG score, stage of lung cancer at diagnosis, PD-L1 expression, and simultaneous chemotherapy-immunotherapy treatment. For survival at 18-months after starting immunotherapy (Table 2), only two covariates (ECOG score and stage of lung cancer at diagnosis) were significant at p < 0.20; however, the decision to include all the covariates from the other “survival” outcome (following lung diagnosis) was made due to clinical considerations. 

**Table 1:** Bivariate comparisons of demographic and clinical variables for survival at 18-months after diagnosis of lung cancer. There was only one patient we did not follow for 18 months after diagnosis – he was diagnosed in November 2021 and alive at the time of data collection (April 2022), with a survival duration of five and nine months after NSCLC diagnosis and starting immunotherapy, respectively. (a) p-value, probability value; (b) aspirin/aggregated immunotherapy, combined aspirin use and immunotherapy of any type; (c) PD-1, programmed cell death-1; (d) PD-L1, programmed death-ligand 1; (e) COPD, chronic obstructive pulmonary disease; (f) ECOG, Eastern Cooperative Oncology Group; (g) NSCLC, non-small-cell lung cancer

	Alive (n = 255)	Deceased (n = 245)	p-value
Combined aspirin/aggregated immunotherapy (n, %)	Yes: (99, 50.3%)	Yes: (98, 49.7%)	0.79
	No: (156, 51.5%)	No: (147, 48.5%)	
Combined aspirin/specific type of immunotherapy (n, %)	Anti-PD-1: (179 ,48.9%)	Anti-PD-1 (187, 51.1%)	0.40
	Anti-PD-L1: (63, 53.4%)	Anti-PD-L1: (55, 46.6%)	
Age (n, %)	21-60 years: (60, 53.6%)	21-60 years: (52,46.4%)	0.22
	61-75 years: (145, 52.9%)	61-75 years: (129, 47.1%)	
	> 75 years: (50, 43.9%)	> 75 years: (64, 56.1%)	
Gender (n, %)	Female: (111, 50.5%)	Female: (109, 49.5%)	0.83
	Male: (144, 51.4%)	Male: (136, 48.6%)	
Race (n, %)	Caucasian: (235, 51.0%)	Caucasian: (226, 49.0%)	0.97
	Non-Caucasian: (20, 51.3%)	Non-Caucasian: (19, 48.7%)	
Pre-existing COPD (n, %)	None or mild: (142, 46.4%)	None or mild: (164, 53.6%)	0.01
	Moderate to very severe: (113, 58.2%)	Moderate to very severe: (81, 41.8%)	
ECOG score (n, %)	ECOG 0: (61, 55.5%)	ECOG 0: (49, 44.5%)	0.02
	ECOG 1: (160, 53.3%)	ECOG 1: (140, 46.7%)	
	ECOG 2-3: (34, 37.8%)	ECOG 2-3: (56, 62.2%)	
NSCLC subtype (n, %)	Adenocarcinoma: (164, 50.6%)	Adenocarcinoma: (160, 49.4%)	0.95
	Squamous cell: (76, 51.4%)	Squamous cell: (72, 48.6%)	
	Other NSCLC: (15, 53.6%)	Other NSCLC: (13, 46.4%)	
Stage of lung cancer (n, %)	Stage I or II: (64, 70.3%)	Stage I or II: (27, 29.7%)	< 0.001
	Stage III: (78, 54.5%)	Stage III: (65, 45.5%)	
	Stage IV: (113, 42.5%)	Stage IV: (153, 57.5%)	
PD-L1expression (n, %)	Negative or unknown: (154, 48.6%)	Negative or unknown: (163, 51.4%)	0.18
	1%-40%: (52, 59.8%)	1%-40%: (35, 40.2%)	
	≥ 41%: (49, 51.0%)	≥ 41%: (47, 49.0%)	
Simultaneous chemotherapy with immunotherapy (n, %)	Yes: (124, 47.0%)	Yes: (140, 53.0%)	0.06
	No: (131, 55.5%)	No: (105, 44.5%)	

**Table 2:** Bivariate comparisons of demographic and clinical variables for survival at 18-months after starting immunotherapy. (a) p-value, probability-value; based on separate Chi-square tests; (b) aspirin/aggregated immunotherapy, combined aspirin use and immunotherapy of any type; (c) PD-1, programmed cell death-1; (d) PD-L1, programmed death-ligand 1; (e) COPD, chronic obstructive pulmonary disease; (f) ECOG, Eastern Cooperative Oncology Group; (g) NSCLC, non-small-cell lung cancer

	Alive (n = 174)	Deceased (n = 326)	p-value
Combined aspirin/aggregated immunotherapy (n, %)	Yes: (63, 32.0%)	Yes: (134, 68.0%)	0.29
	No: (111, 36.6%)	No: (192, 63.4%)	
Combined aspirin/specific type of immunotherapy (n, %)	Anti-PD-1: (121, 33.1%)	Anti-PD-1: (246, 66.9%)	0.61
	Anti-PD-L1: (42, 35.6%)	Anti-PD-L1: (76, 64.4%)	
Age (n, %)	21-60 years: (44, 39.3%)	21-60 years: (68, 60.7%)	0.40
	61-75 years: (95, 34.7%)	61-75 years: (179, 65.3%)	
	> 75 years: (35, 30.7%)	> 75 years: (79, 69.3%)	
Gender (n, %)	Female: (74, 33.6%)	Female: (146, 66.4%)	0.63
	Male: (100, 35.7%)	Male: (180, 64.3%)	
Race (n, %)	Caucasian: (158, 34.3%)	Caucasian: (303, 65.7%)	0.40
	Non-Caucasian: (16, 41.0%)	Non-Caucasian: (23, 59.0%)	
Pre-existing COPD (n, %)	None or mild: (108, 35.3%)	None or mild: (198, 64.7%)	0.77
	Moderate to very severe: (66, 34.0%)	Moderate to very severe: (128, 66.0%)	
ECOG score (n, %)	ECOG 0: (45, 40.9%)	ECOG 0: (65, 59.1%)	< 0.001
	ECOG 1: (114, 38.0%)	ECOG 1: (186, 62.0%)	
	ECOG 2-3: (15, 16.7%)	ECOG 2-3: (75, 83.3%)	
NSCLC subtype (n, %)	Adenocarcinoma: (119, 36.7%)	Adenocarcinoma: (205, 63.3%)	0.47
	Squamous cell: (46, 31.1%)	Squamous cell: (102, 68.9%)	
	Other NSCLC: (9, 32.1%)	Other NSCLC: (19, 67.9%)	
Stage of lung cancer (n, %)	Stage I or II: (31, 34.1%)	Stage I or II: (60, 65.9%)	0.15
	Stage III: (59, 41.3%)	Stage III: (84, 58.7%)	
	Stage IV: (84, 31.6%)	Stage IV: (182, 68.4%)	
PD-L1 expression (n, %)	Negative or unknown: (102, 32.2%)	Negative or unknown: (215, 67.8%)	0.27
	1-40%: (34, 39.1%)	1-40%: (53, 60.9%)	
	≥ 41%: (38, 39.6%)	≥41%: (58, 60.4%)	
Simultaneous chemotherapy with immunotherapy (n, %)	Yes: (87, 33.0%)	Yes: (177, 67.0%)	0.36
	No: (87, 36.9%)	No: (149, 63.1%)	

Neither combined ASA/aggregated immunotherapy treatment nor combined ASA/specific type of immunotherapy (PD-1 vs. PD-L1 inhibitors) was significantly different following bivariate analysis; therefore, they were not included in these two “survival” models.

For CR displayed in Table 3, the following covariates were included in the regression model (p < 0.10): combined ASA/specific type of immunotherapy, pre-existing COPD, ECOG score, stage of lung cancer at diagnosis, and simultaneous chemotherapy-immunotherapy treatment. Finally, for progressive disease (PD), the following covariates were included in the regression model (p < 0.20): combined ASA/specific type of immunotherapy, gender, ECOG score, and type of lung cancer (Table 4).

**Table 3:** Bivariate comparisons of demographic and clinical variables for complete remission (as defined by RECIST criteria). (a) p-value, probability-value; based on separate Chi-square tests; (b) aspirin/aggregated immunotherapy, combined aspirin use and immunotherapy of any type;  (c) PD-1, programmed cell death-1; (d) PD-L1, programmed death-ligand 1; (e) COPD, chronic obstructive pulmonary disease; (f) ECOG, Eastern Cooperative Oncology Group; (g) NSCLC, non-small-cell lung cancer

	Remission (n = 417)	No remission (n = 83)	p-value
Combined aspirin/aggregated immunotherapy (n, %)	Yes: (31, 15.7%)	Yes: (166, 84.3%)	0.68
	No: (52, 17.2%)	No: (251, 82.8%)	
Combined aspirin/specific type of immunotherapy (n, %)	Anti-PD-1: (43, 11.7%)	Anti-PD1: (323, 88.3%)	< 0.001
	Anti-PD-L1: (36, 30.5%)	Anti-PDL1: (82, 69.5%)	
Age (n, %)	21-60 years: (21, 18.8%)	21-60 years: (91, 81.3%)	0.74
	61-75 years: (45, 16.4%)	61-75 years: (229, 83.6%)	
	> 75 years: (17, 14.9%)	> 75 years: (97, 85.1%)	
Gender (n, %)	Female: (35, 15.9%)	Female: (185, 84.1%)	0.71
	Male: (48, 17.1%)	Male: (232, 82.9%)	
Race (n, %)	Caucasian: (74, 16.1%)	Caucasian: (387, 83.9%)	0.26
	Non-Caucasian: (9, 23.1%)	Non-Caucasian: (30, 76.9%)	
Pre-existing COPD (n, %)	None or mild: (40, 13.1%)	None or mild: (266, 86.9%)	0.008
	Moderate to very severe: (43, 22.2%)	Moderate to very severe: 151 (77.8%)	
ECOG score (n, %)	ECOG 0: (22, 20.0%)	ECOG 0: (88, 80.0%)	0.02
	ECOG 1: (55, 18.3%)	ECOG 1: (245, 81.7%)	
	ECOG 2-3: (6, 6.7%)	ECOG 2-3: (84, 93.3%)	
NSCLC^(g)^ subtype (n, %)	Adenocarcinoma: (52, 16.0%)	Adenocarcinoma: (272, 84.0%)	0.79
	Squamous cell: (27, 18.2%)	Squamous cell: (121, 81.8%)	
	Other NSCLC: (4, 14.3%)	Other NSCLC: (24, 85.7%)	
Stage of lung cancer (n, %)	Stage I or II: (25, 27.5%)	Stage I or II: (66, 72.5%)	< 0.001
	Stage III: (34, 23.8%)	Stage III: (109, 76.2%)	
	Stage IV: (24, 9.0%)	Stage IV: (242, 91.0%)	
PD-L1 expression (n, %)	Negative or unknown: (55, 17.4%)	Negative or unknown: (262, 82.6%)	0.29
	1%-40%: (17, 19.5%)	1-40%: (70, 80.5%)	
	≥ 41%: (11, 11.5%)	≥ 41%: (85, 88.5%)	
Simultaneous chemotherapy with immunotherapy (n, %)	Yes: (30, 11.4%)	Yes: (234, 88.6%)	< 0.001
	No: (53, 22.5%)	No: (183, 77.5%)	

**Table 4:** Bivariate comparisons of demographic and clinical variables for PD (as defined per RECIST criteria). (a) p-value, probability value; based on separate Chi-square tests; (b) aspirin/aggregated immunotherapy, combined aspirin use and immunotherapy of any type; (c) PD-1, programmed cell death-1; (d) PD-L1, programmed death-ligand 1; (e) COPD, chronic obstructive pulmonary disease; (c) ECOG, Eastern Cooperative Oncology Group; NSCLC, non-small cell lung cancer PD, progressive disease

	PD (n = 198)	Non-PD (n = 302)	p-value
Combined aspirin/aggregated immunotherapy (n, %)	Yes: (78, 39.6%)	Yes: (119, 60.4%)	1.00
	No: (120, 39.6%)	No: (183, 60.4%)	
Combined aspirin/specific type of immunotherapy (n, %)	Anti-PD-1: (160, 43.7%)	Anti-PD-1: (206, 56.3%)	0.001
	Anti-PD-L1: (32, 27.1%)	Anti-PD-L1: (86, 72.9%)	
Age (n, %)	21-66 years: (45, 40.2%)	21-66 years: (67, 59.8%)	0.89
	61-75 years: (106, 38.7%)	61-75 years: (168, 61.3%)	
	> 75 years: (47, 41.2%)	> 75 years: (67, 58.8%)	
Gender (n, %)	Female: (77, 35.0%)	Female: (143, 65.0%)	0.06
	Male: (121, 43.2%)	Male: (159, 56.8%)	
Race (n, %)	Caucasian: (181, 39.3%)	Caucasian: (280, 60.7%)	0.60
	Non-Caucasian: (17, 43.6%)	Non-Caucasian: (22, 56.4%)	
Pre-existing COPD (n, %)	None or mild: (121, 39.5%)	None or mild: (185, 60.5%)	0.97
	Moderate to very severe: (77, 39.7%)	Moderate to very severe: (117, 60.3%)	
ECOGscore (n, %)	ECOG 0: (39, 35.5%)	ECOG 0: (71, 64.5%)	0.01
	ECOG 1: (111, 37.0%)	ECOG 1: (189, 63.0%)	
	ECOG 2-3: (48, 53.3%)	ECOG 2-3: (42, 46.7%)	
Type of lung cancer (n, %)	Adenocarcinoma: (119, 36.7%)	Adenocarcinoma: (205, 63.3%)	0.03
	Squamous cell: (71, 48.0%)	Squamous cell: (77, 52.0%)	
	Other NSCLC: (8, 28.6%)	Other NSCLC: (20, 71.4%)	
Stage of lung cancer (n, %)	Stage I or II: (37, 40.7%)	Stage I or II: (54, 59.3%)	0.40
	Stage III: (50, 35.0%)	Stage III: (93, 65.0%)	
	Stage IV: (111, 41.7%)	Stage IV: (155, 58.3%)	
PD-L1 expression (n, %)	Negative or unknown: (121, 38.2%)	Negative or unknown: 196, 61.8%)	0.38
	1-40%: (33, 37.9%)	1-40%: (54, 62.1%)	
	≥ 41%: (44, 45.8%)	≥ 41%: (52, 54.2%)	
Simultaneous chemotherapy with immunotherapy (n, %)	Yes: (100, 37.9%)	Yes: (164, 62.1%)	0.41
	No: (98, 41.5%)	No: (138, 58.5%)	

As shown in Table 5 (survival at 18-months after diagnosis of lung cancer), the model had adequate goodness-of-fit based on the omnibus chi-square and Hosmer-Lemeshow p-values. However, the overall correct classification rate was only 60.4% based on the included covariates. The following covariates were independently and significantly associated with a decreased likelihood of survival: ECOG score of 2-3 (AOR = 0.44), Stage III cancer (AOR = 0.54), and Stage IV cancer (AOR = 0.37). Both pre-existing COPD (AOR = 1.41) and low PD-L1 expression (AOR = 1.53) showed a trend toward an increased likelihood of survival, but neither had statistical significance (p = 0.09 and 0.10, respectively).

**Table 5:** Multivariable logistic regression for survival at 18-months after diagnosis of lung cancer. (a) AOR, adjusted odds ratio; CI, confidence interval. (b) p-value, probability-value; Omnibus Chi-square p-value < 0.001; Hosmer-Lemeshow goodness-of-fit p-value = 0.42; 60.4% overall correct classification rate. (c) COPD, chronic obstructive pulmonary disease; (d) ECOG, Eastern Cooperative Oncology Group; (e) PD-L1: programmed death-ligand 1

Covariates	AOR (95% CI)	p-value
Pre-existing COPD	1.41 (0.95 - 2.09)	0.09
ECOG score (reference = ECOG 0)	ECOG 1: 0.86 (0.55 - 1.36)	0.53
	ECOG 2-3: 0.44 (0.24 - 0.80)	0.007
Stage of lung cancer (reference = Stage I or II)	Stage III: 0.54 (0.31 - 0.97)	0.04
	Stage IV: 0.37 (0.22 - 0.64)	< 0.001
PD-L1 expression (reference = negative or unknown)	1%-40%: 1.53 (0.92 - 2.53)	0.10
	≥ 41%: 1.24 (0.76 - 2.01)	0.39
Simultaneous chemotherapy with immunotherapy	0.79 (0.53 - 1.17)	0.24

The model shown in Table 6 (survival at 18-months after starting immunotherapy) had adequate goodness-of-fit based on the omnibus chi-square and Hosmer-Lemeshow p-values. However, the overall correct classification rate was only 63.2% based on the included covariates. Only an ECOG score of 2-3 (AOR = 0.28) was independently and significantly associated with a decreased likelihood of survival.

**Table 6:** Multivariable logistic regression for survival at 18-months after starting immunotherapy. (a) AOR, adjusted odds ratio; CI, confidence interval. (b) p-value, probability-value; Omnibus Chi-square p-value = 0.001; Hosmer-Lemeshow goodness-of-fit p-value = 0.13; 63.2% overall correct classification rate. (c) COPD, chronic obstructive pulmonary disease; (d) ECOG, Eastern Cooperative Oncology Group; (e) PD-L1, programmed death-ligand 1

Covariates	AOR (95% CI)	p-value
Pre-existing COPD (reference = none or mild)	1.00 (0.66 – 1.51)	0.99
ECOG score (reference = ECOG 0)	ECOG 1: 0.85 (0.54 – 1.34)	0.49
	ECOG 2-3: 0.28 (0.14 - 0.55)	< 0.001
Stage of lung cancer (reference = Stage I or II)	Stage III: 1.47 (0.83 – 2.61)	0.19
	Stage IV: 0.94 (0.54 – 1.63)	0.83
PD-L1 expression (reference = negative or unknown)	1%-40%: 1.38 (0.83 – 2.28)	0.22
	≥ 41%: 1.52 (0.92 – 2.50)	0.10
Simultaneous chemotherapy with immunotherapy	0.90 (0.60 – 1.36)	0.63

As indicated in the footnote for Table 7 (CR), the model had adequate goodness-of-fit based on the omnibus chi-square and Hosmer-Lemeshow p-values. Based on the included covariates, the overall correct classification rate was 83.3%. The following covariates were independently and significantly associated with a decreased likelihood of CR: ECOG score of 2-3 (AOR = 0.25) and Stage IV cancer (AOR = 0.34). Although PD-L1 immunotherapy combined with ASA was associated with a higher likelihood of CR (AOR = 1.85), it was not statistically significant (p = 0.06).

**Table 7:** Multivariable logistic regression for complete remission. (a) AOR, adjusted odds ratio; CI: confidence interval. (b) p-value, probability-value; Omnibus Chi-square p-value < 0.001; Hosmer-Lemeshow goodness-of-fit p-value = 0.68; 83.3% overall correct classification rate; (c) PD-1, programmed cell death-1; (d) COPD, chronic obstructive pulmonary disease; (e) ECOG, Eastern Cooperative Oncology Group

Covariates	AOR (95% CI)	p-value
Combined aspirin/specific type of immunotherapy (reference = PD-1)	1.85 (0.99 - 3.46)	0.06
Pre-existing COPD (reference = none or mild)	1.37 (0.80 - 2.36)	0.25
ECOGscore (reference = E0)	ECOG 1: 0.74 (0.41 - 1.36)	0.34
	ECOG 2-3: 0.25 (0.09 - 0.68)	0.007
Stage of lung cancer (reference = Stage I or II)	Stage III: 0.62 (0.31 - 1.24)	0.18
	Stage IV: 0.34 (0.17 - 0.67)	0.001
Simultaneous chemotherapy with immunotherapy	0.60 (0.33 - 1.08)	0.09

As indicated in the footnote for Table 8 (PD), the model had adequate goodness-of-fit based on the omnibus chi-square and Hosmer-Lemeshow p-values. However, the overall correct classification rate was 61.8% based on the included covariates. Only an ECOG score of 2-3 (AOR = 1.88) was independently and significantly associated with an increased likelihood of PD. PD-L1 (not PD-1) immunotherapy combined with ASA (AOR = 0.44) was independently associated with a decreased likelihood of PD, with remarkably high statistical significance (p < 0.001). Although the male gender had higher odds of having PD (AOR = 1.45), it did not reach statistical significance (p = 0.06).

**Table 8:** Multivariable logistic regression for PD. (a) AOR, adjusted odds ratio; CI, confidence interval; (b) p-value, probability-value; Omnibus Chi-square p-value < 0.001; Hosmer-Lemeshow goodness-of-fit p-value = 0.79; 61.8% overall correct classification rate. (c) PD-1, programmed cell death-1; (d) ECOG, Eastern Cooperative Oncology Group; (e) NSCLC, non-small cell lung cancer PD, progressive disease

Covariates	AOR (95% CI)	p-value
Combined aspirin/specific type of immunotherapy (reference = PD-1)	0.44 (0.27 - 0.71)	< 0.001
Gender (reference = female)	1.45 (0.99 - 2.13)	0.06
ECOG score (reference = ECOG 0)	ECOG 1: 1.05 (0.65 - 1.70)	0.84
	ECOG 2-3: 1.88 (1.04 - 3.42)	0.04
Type of lung cancer (reference = NSCLC)	Adenocarcinoma: 1.08 (0.45 - 2.60)	0.87
	Squamous cell: 1.87 (0.75 - 4.65)	0.18

Tables 9-10 present the results of the secondary outcomes. Combined ASA/aggregated immunotherapy patients had slightly worse adverse effects, but none of the between-group differences were statistically significant. Compared to PD-1 immunotherapy with ASA, patients who used ASA with PD-L1 inhibitors had significantly higher rates of pneumonitis (37.3% vs. 24.0%, p = 0.005). However, none of the other adverse events were significantly different. To further analyze the difference in pneumonitis incidence and severity between the two groups (ASA-users treated with PD-1 vs. PD-L1 inhibitors), pneumonitis grades were determined according to the National Cancer Institute CTCAE v5.0 Criteria (Table 11). 

**Table 9:** Secondary outcomes for combined aspirin and immunotherapy (aggregated). p-value, probability value; based on the Chi-square test

	Pneumonitis (n, %)	Hematologic complications (n, %)	Gastroenteric complications (n, %)	Other complications (n, %)
Aspirin with immunotherapy (n = 197)	(57, 28.9%)	(72, 36.5%)	(73, 37.1%)	(56, 28.4%)
No-aspirin with immunotherapy (n = 303)	(81, 26.7%)	(95, 31.4%)	(110, 36.3%)	(82, 27.1%)
p-value	0.59	0.23	0.87	0.74

**Table 10:** Secondary outcomes for combined aspirin and specific immunotherapy (separated by type). (a) PD-1: programmed cell death-1; (b) PD-L1: programmed death-ligand 1; (c) p-value: probability value; based on the Chi-square test.

	Pneumonitis (n, %)	Hematologic complications (n, %)	Gastroenteric complications (n, %)	Other complications (n, %)
PD-1 inhibitor with aspirin (n = 366)	(88, 24.0%)	(127, 34.7%)	(129, 35.2%)	(101, 27.6%)
PD-L1 inhibitor with aspirin (n = 118)	(44, 37.3%)	(33, 28.0%)	(49, 41.5%)	(30, 25.4%)
p-value	0.005	0.18	0.22	0.64

**Table 11:** Pneumonitis grades in aspirin users treated with PD-1 vs. PD-L1 inhibitors. (a) PD-1, programmed cell death-1; (b) PD-L1, programmed death-ligand 1

Pneumonitis cases and grade	I (n, %)	II (n, %)	III (n, %)	IV (n, %)	V (n, %)
PD-1 inhibitor with aspirin (n = 88)	18 (20.4%)	20 (22.7%)	41 (46.6%)	9 (10.2%)	0 (0%)
PD-L1 inhibitor with aspirin (n = 44)	13 (29.5%)	14 (27.3%)	12 (27.3%)	5 (11.4%)	0 (0%)

## Discussion

**Figure 2:**
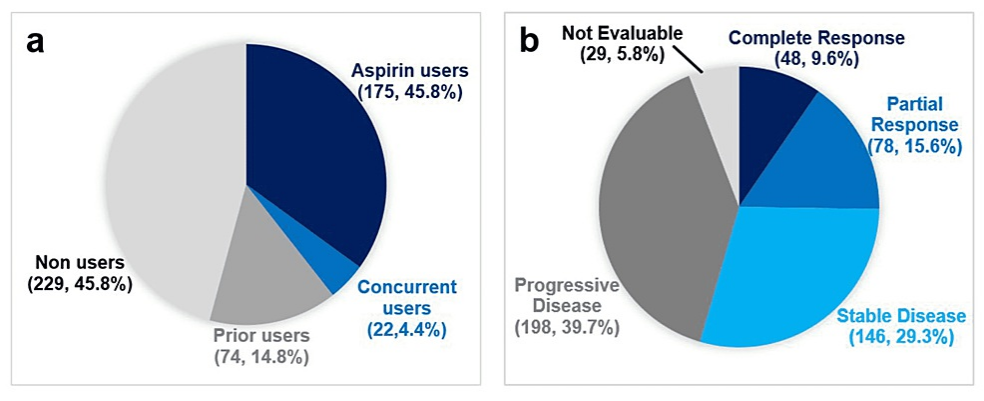
(a) Aspirin use (n, %); (b) RECIST (n, %). Aspirin users: 81 mg daily before and with immunotherapy; concurrent users: started daily aspirin with and while receiving immunotherapy; prior users: used in the past, but not with immunotherapy; non-users: no daily aspirin use. RECIST, Response Evaluation Criteria in Solid Tumors. Modified from our REDCap data analysis report.

One of the primary outcomes of our study was to assess whether ASA use with immunotherapy increases NSCLC patients' survival. This hypothesis was based on prior findings of both in vitro and human studies, as noted above [1-6]. By inhibiting the cyclooxygenase II enzyme, ASA decreases the production of PGE-2, which reduces tumor-derived inflammation and growth. The bivariate analyses found no statistical association between ASA use (with immunotherapy) and survival. Therefore, combined ASA/immunotherapy was not included in the subsequent regression modeling (Table 12). The lack of significance here could be due to the limited number of ASA users (including those who started ASA while on immunotherapy) in our sample (197, 39.4%) compared to others who did not use ASA with immunotherapy (303, 60.6%) (Figure 2a). A more extensive retrospective study of 9,864 non-operable NSCLC patients, 4,979 (50.5%) of them were ASA users, suggested that ASA use was associated with more prolonged overall survival (OS), with a median OS of 1.73 years compared to 1.30 years of the non-ASA users [12]. However, it did not address the type of immunotherapy used (if any) with the ASA.

**Table 12:** Summary of the remarkable findings and associations. (a) AOR, adjusted odds ratio; (b) CI, confidence interval; (c) p-value, probability value; †, close to but not statistically significant; (d) PD, progressive disease; (e) ECOG, Eastern Cooperative Oncology Group – Performance Status; (f) 18M S-D: survival at 18-months after cancer diagnosis; (g) 18M S-I: survival at 18-months after starting immunotherapy; (h) CR, complete remission; (i) COPD, chronic obstructive pulmonary disease; (k) NSCLC, non-small cell lung cancer; (m) PD-L1, programmed death-ligand 1

Variables		Association: decreased (↓) or increased (↑); none (-)	Outcome	AOR	95% CI	p-value^† ^
Patient-related	Male gender	↑	PD	1.45	(0.99-2.13)	0.06†
	ECOG 2 or 3	↓	18M S-D	0.44	(0.24-0.80)	0.007
		↓	18M S-I	0.28	(0.14-0.55)	< 0.001
		↓	CR	0.25	(0.09-0.68)	0.007
		↑	PD	1.88	(1.04-3.42)	0.04
	COPD moderate to very severe	↑	18M S-D	1.41	(0.95-2.09)	0.09†
Cancer-related	NSCLCStage III	↓	18M S-D	0.54	(0.31-0.97)	0.04
	NSCLC Stage IV	↓	18M S-D	0.37	(0.22-0.64)	< 0.001
		↓	CR	0.34	(0.17-0.67)	0.001
	Low PD-L1 expression	↑	18M S-D	1.53	(0.92-2.53)	0.10†
Therapy-related	Combined aspirin with immunotherapy	-	18M S-D	-	-	-
		-	18M S-I	-	-	-
	Combined aspirin with PD-L1	↑	CR	1.85	(0.99-3.46)	0.06†
		↓	PD	0.44	(0.27-0.71)	< 0.001
	Chemotherapy with immunotherapy	↓	CR	0.60	(0.33-1.08)	0.09†

On the other hand, another study compared PD-1 and PD-L1 inhibitors in patients with cancer and suggested a more remarkable OS with anti-PD1 [13]. Still, again it did not address the use of ASA with either immunotherapy type, or it did not focus on lung cancer. Further studies are needed to assess the effect of ASA use on the OS of NSCLC patients receiving immunotherapy. 

To the best of our knowledge, this is one of the first studies to investigate the association between ASA use with distinct types of immunotherapies and the likelihood of achieving remission versus PD after NSCLC diagnosis (Figure 2b). The daily use of ASA with PD-L1 inhibitors was significantly and independently associated with decreased odds of having the PD (AOR = 0.44) than PD-1 with ASA. Similarly, daily ASA use with PD-L1 inhibitors showed a trend toward achieving CR but only with a probability close to significance (AOR = 1.85, 95% CI 0.99-3.46, p = 0.06). As was noted before, in vitro studies suggested a synergic effect of adding NSAID/ASA to PD-1/ PD-L1 inhibitors for a better anti-cancer effect [5-7]. More specifically, researchers found that ASA suppressed the growth of cancer cells, including lung cancer cells, via inhibiting PDL-1 overexpression [14-15]. Those findings could explain our suggested clinical benefit of using ASA in NSCLC patients at biochemical and histological levels, especially with PD-L1 inhibitors.

Still, there was no previous comparison regarding the likelihood of achieving remission vs. PD in the two groups (PD-1 and PD-L1 inhibitors) combined with ASA. Other studies focused on the (overall and progression-free) survival rate as the main outcome, not the response of the solid tumors. While many others evaluated ASA’s effect on non-lung cancers, and few investigated its effect on lung cancer, they were either limited to advanced NSCLC or did not compare the effect in different immunotherapy groups [16]. Further studies are needed to explore this suggested effect; if proven clinically significant, ASA could be incorporated into the treatment regimen for NSCLC patients (especially with PD-L1 inhibitors) if not otherwise contraindicated.

**Figure 3:**
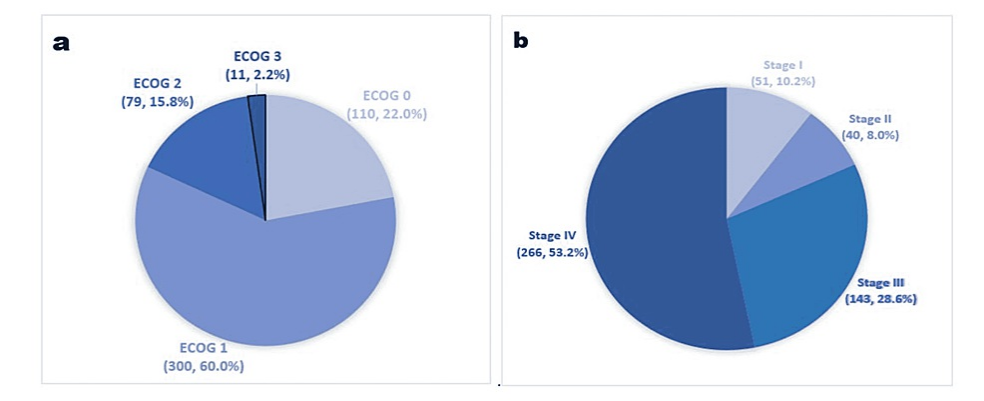
(a) ECOG score at the time of lung cancer diagnosis (n, %); (b) NSCLC stage at diagnosis (n, %). ECOG, Eastern Cooperative Oncology Group; ECOG 4 and 5 were not included since we did not have any patients with an ECOG of 4 or 5. Modified from our REDCap data analysis report. NSCLC, non-small cell lung cancer

Our data confirm prior associations between poorer PS and shorter survival. This work may be the first to report on an association between PS and the likelihood of achieving CR vs. PD, as defined by RECIST criteria. Poorer PS at the time of diagnosis (ECOG of 2 or 3) was significantly and independently associated with decreased odds of survival at 18-months (after diagnosis, AOR = 0.44, and after immunotherapy, AOR = 0.28). It was also associated with reduced odds of achieving CR (AOR = 0.25) and an increased likelihood of having a PD (AOR = 1.88) compared to ECOG of 1 (Figure 3a). A prior study of 1,655 cancer patients concluded that using PS (ECOG) as a prognostic tool for survival in advanced cancer was as effective as using more complex models like the Palliative Performance Scale and Karnofsky Performance Scale [17]. Another study of 1,868 primary lung cancer patients found that an ECOG of 3 or higher was associated with lower survival [18]. Given these findings, we emphasize evaluating PS using ECOG at diagnosis and incorporating it into shared decision-making and prognosis of patients with NSCLC.

Another important variable associated with the study's primary outcomes was the advanced stage of NSCLC at diagnosis (Figure 3b). Both stages III and IV at diagnosis of NSCLC were significantly and independently associated with a decreased likelihood of survival at 18 months after diagnosis (AOR = 0.54 and 0.37, respectively). Only Stage IV (not III) was associated with reduced odds of achieving CR (AOR = 0.34). These findings confirm a well-established relationship between advanced NSCLC stage and poorer survival [19-20]. Still, we addressed a more specific outcome - the likelihood of achieving CR.

**Figure 4:**
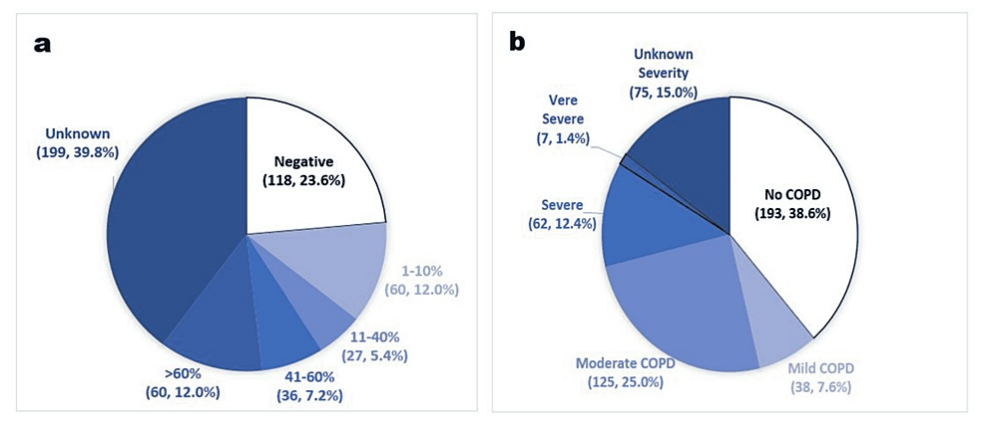
(a) PD-L1 expression (n, %); (b) chronic obstructive pulmonary disease severity (n, %). Modified from our REDCap data analysis report. PD-L1, programmed death-ligand 1

The NSCLC-PD-L1 low expression (1%-40%) had a trend towards increased odds of 18-month survival after diagnosis (AOR = 1.53, 95% CI 0.92-2.53) but without significant probability (p = 0.10). The lack of statistical significance could be due to the limited sample size, especially with 199 (39.8%) of our 500 patients having unknown (not tested) PDL-1 expression status (Figure 4a). However, it may be of clinical importance if this trend is reproduced and confirmed in future studies. A more extensive retrospective study of 617 patients with CRC and known PD-L1 expression status found a stronger association between ASA use and CRC survival in PD-L1-low tumors [21]. More studies are needed to evaluate a similar potential benefit of ASA use in NSCLC, especially with low PD-L1 expression and immunotherapy.

Pre-existing COPD (moderate to very severe) showed a trend toward an increased likelihood of survival at 18-months after diagnosis, but it failed to reach statistical significance (p = 0.09). Some studies suggested an association between COPD and poorer survival in early-stage NSCLC [22-23]. In contrast, others that focused on only advanced stages of NSCLC (IIIb-IV) at diagnosis found no significant deleterious impact of COPD on survival [24]. A suggested explanation for these contradictory findings is that the negative effect of COPD on survival is more evident in patients with early-stage NSCLC than in advanced disease [25-26]. Most of the patients included in our study (409 of 500) had advanced NSCLC at the time of diagnosis (Figure 4b), which may explain our finding of improved survival odds in patients with moderate to very severe COPD. Additional studies are needed to examine the effect of COPD severity on the survival of patients with different NSCLC stages. At the same time, we wish to emphasize relevant previous findings that undiagnosed or undertreated COPD in NSCLC patients resulted in poorer quality of life and worse outcomes [25, 27].

Our findings suggest that the male gender could be associated with poorer outcomes, particularly the increased likelihood of PD after NSCLC diagnosis. While close to but not statistically significant in our study (p = 0.06), it is consistent with prior findings, including a cohort of 4,618 patients and a large meta-analysis involving 85,800 patients [28-29].

We found no significant difference between the ASA and non-ASA groups for the secondary outcomes: therapy-related adverse effects and toxicities. However, among ASA users, patients treated with PD-L1 inhibitors had a higher pneumonitis incidence than those treated with PD-1 inhibitors (37.3% vs. 24.0%, respectively). This finding is not consistent with the incidence rates reported in previous studies [30]. However, when considering the degree of severity, a more considerable proportion of the pneumonitis cases in the PD-1 group were of grade 3 or higher than those in the PD-L1 group (56.8% vs. 38.6%, respectively). Further studies are needed to compare the incidence and severity of pneumonitis between the two groups (PD-1 and PD-L1) and to clarify whether the use of ASA makes any significant difference.

Our study was limited to a single-center population, and therefore it might not present the entire population. The non-Caucasian population was underrepresented in our sample given other races combined formed only 7.8% of our population sample. Although the overall sample size was sufficient to conduct the study, some subgroups did not have enough sample sizes and, therefore, were either excluded or combined together. Examples of the subgroups lacking adequate sample sizes included the large cell carcinoma (combined with other types of NSCLC than adenocarcinoma and SCC), lung cancer stages I and II (combined together), and immunotherapies other than PD-1/PD-L1 inhibitors (were excluded). Although we addressed concurrent treatment with chemotherapy and immunotherapy as a covariant and did not have a significant association with our study's outcomes (except in the bivariate analysis for CR), we did not investigate/compare the different types of chemotherapy agents used. 

Additionally, being a retrospective study has its own intrinsic limitations such as dependence on previous data and documentation. Our study used a binary model to determine ASA usage (minimum 81 mg daily), but it may be beneficial to further assess the length of ASA usage in future studies to determine if there is a temporal or dose-response to ASA in the context of immunotherapy. For the same reason, the nature of the study, many of our patients (n=199), did not have a PD-L1 expression status available in their records (mainly because it was not tested). 

## Conclusions

We found that the daily use of ASA combined with immunotherapy, particularly PD-L1 inhibitors, was associated with better outcomes in patients with NSCLC. ASA users who received PD-L1 inhibitors (Durvalumab or Atezolizumab) were more likely to achieve CR and had lower odds of having PD. Our study emphasizes using the ECOG score as an effective predictive tool for survival and response to therapy (remission vs. PD) in NSCLC patients. Poorer PS (an ECOG of 2 or 3) and advanced cancer stage (III or IV) were associated independently with unfavorable outcomes in NSCLC patients treated with immunotherapy. Learning more about the potential prognostic and risk factors in NSCLC can help us provide better treatments, more accurate prognoses, and achieve optimal outcomes.
